# Unsuspected Dengue as a Cause of Acute Febrile Illness in Children and Adults in Western Nicaragua

**DOI:** 10.1371/journal.pntd.0005026

**Published:** 2016-10-28

**Authors:** Megan E. Reller, Aravinda M. de Silva, Jeremy J. Miles, Ramesh S. Jadi, Anne Broadwater, Katie Walker, Christopher Woods, Orlando Mayorga, Armando Matute

**Affiliations:** 1 Division of Infectious Diseases, Department of Medicine, Johns Hopkins University School of Medicine, Baltimore, Maryland, United States of America; 2 Hubert-Yeargan Center for Global Health, Durham, North Carolina, United States of America; 3 Department of Microbiology and Immunology, University of North Carolina School of Medicine, Chapel Hill, North Carolina, United States of America; 4 Duke University School of Medicine, Durham, North Carolina, United States of America; 5 Division of Infectious Diseases, Department of Medicine, Duke University School of Medicine, Durham, North Carolina, United States of America; 6 Hospital Escuela Oscar Danilo Rosales Arguello, Universidad Nacional Autonoma de Nicaragua, León, Nicaragua; University of California, Berkeley, UNITED STATES

## Abstract

**Background:**

Dengue is an emerging infectious disease of global significance. Suspected dengue, especially in children in Nicaragua’s heavily-urbanized capital of Managua, has been well documented, but unsuspected dengue among children and adults with undifferentitated fever has not.

**Methodology/Principal Findings:**

To prospectively study dengue in semi-urban and rural western Nicaragua, we obtained epidemiologic and clinical data as well as acute and convalescent sera (2 to 4 weeks after onset of illness) from a convenience sample (enrollment Monday to Saturday daytime to early evening) of consecutively enrolled patients (n = 740) aged ≥ 1 years presenting with acute febrile illness. We tested paired sera for dengue IgG and IgM and serotyped dengue virus using reverse transcriptase-PCR. Among 740 febrile patients enrolled, 90% had paired sera. We found 470 (63.5%) were seropositive for dengue at enrollment. The dengue seroprevalance increased with age and reached >90% in people over the age of 20 years. We identified acute dengue (serotypes 1 and 2) in 38 (5.1%) patients. Only 8.1% (3/37) of confirmed cases were suspected clinically.

**Conclusions/Significance:**

Dengue is an important and largely unrecognized cause of fever in rural western Nicaragua. Since Zika virus is transmitted by the same vector and has been associated with severe congenital infections, the population we studied is at particular risk for being devastated by the Zika epidemic that has now reached Central America.

## Author Summary

Dengue is an emerging infectious disease of global significance. Unsuspected dengue among children and adults presenting with undifferentiated fever in western Nicaragua has not been studied. We prospectively studied patients ≥ 1 year of age who presented with acute febrile illness in Nicaragua and systematically collected detailed information about exposures and features of the illness as well as serum to confirm acute infections. Overall, 470 (63.5%) had evidence of prior infection with dengue virus; the proportion with antibodies against dengue virus increased with age and reached >90% in those >20 years. Thirty-eight (5.1%) had acute dengue (serotypes 1 and 2) infection. Only 8.1% (3/37) cases were suspected clinically to be dengue. Dengue is an important and largely unrecognized cause of fever in rural western Nicaragua and strikes predominantly those in child-bearing years, the same individuls at risk for devastating complications associated with Zika virus infection that is also transmitted by the *Aedes aegypti* mosquito.

## Introduction

Dengue is an emerging cause of acute febrile illness (AFI) worldwide. Dengue virus (DENV) with its 4 antigenically distinct serotypes is the most common arbovirus worldwide with an estimated ~400 million symptomatic cases occurring annually in over 100 countries [1]. Clinical manifestations of dengue range from asymptomatic or mild acute febrile illness (AFI) to circulatory failure and death from dengue hemorrhagic fever (DHF). Urbanization, rapid movement of humans facilitated by modern transportation and other socioeconomic factors, and geographic expansion of dengue’s primary vector, *Aedes aegypti*, have fueled the current global dengue pandemic [2].

Widespread dengue was first identified in Nicaragua in 1985 when DENV-1 and DENV- 2 caused an outbreak that resulted in over 17,000 cases and seven deaths [3]. Public health measures to control *Aedes aegypti* were effective for nearly a decade with only sporadic outbreaks of DENV-1, DENV-2, and DENV-4 occurring until reintroduced DENV-3 caused a massive outbreak in 1995 [4]. Over the past two decades only one of the 4 dengue serotypes has predominated each season in Nicaragua [5–9]. Data from the Ministry of Health and an ongoing longitudinal cohort study of dengue infections in children in urban Managua [10] suggest that dengue transmission peaks during the rainy season (especially August-November) but occurs sporadically year-round [11]. Since serologic cross-reactions are expected among flaviviruses, epidemiological studies of dengue in Nicaragua have been facilitated by lack of reports of yellow fever and Japanese encephalitis, a single report of West Nile virus in a traveler from Spain [12], and the introduction of Zika virus only recently [13].

To ascertain the role of dengue as a cause of AFI we studied consequtively enrolled patients ≥ 1 year of age who presented with acute febrile illness to a large teaching hospital in León, Nicaragua, which has a population of ~210,000 and is located along the Río Chiquito 90 kilometres northwest of Managua and ~ 18 km east of the Pacific Ocean. Although León is Nicaragua's second largest city, the districts surrounding León are relatively rural. The study was conducted over 10 months from August 2008 to May 2009.

## Methods

### Ethics statement

Study doctors verified eligibility and willingness to return for a 2 to 4 week convalescent follow-up visit and obtained written informed consent from adult patients (≥18 years) or parents of pediatric (<18 years) patients along with assent if aged 12 to 17 years. The institutional review boards of Johns Hopkins University (NA_00012415), Duke University Medical Center (Pro00014461), and Universidad Nacional Autonoma de Nicaragua (00003242), León (Nicaragua) approved the study and the University of North Carolina (UNC) approved use of samples (UNC deemed study exempt from IRB review).

### Setting and Patients

We recruited patients in the emergency department and adult and pediatric wards of the Hospital Escuela Oscar Danilo Rosales Arguello (HEODRA), the 400-bed primary public teaching hospital of Universidad Nacional Autonoma de Nicaragua (UNAN) in León, Nicaragua. Between August 2008 and May 2009, we enrolled consecutive consenting febrile (≥38°C, tympanic) patients ≥1 year old without prior (within 1 week) trauma or hospitalization who presented during the day or early evening hours Monday through Saturday. On enrollment (single point in time), study personnel recorded on a standardized form epidemiological information, including self-reported urban vs. rural residence and exposures, and clinical features, including duration of illness, history of present illness, findings on examination, and the clinical provider’s presumptive diagnosis. Additionally, specimens for on-site clinician-requested testing and off-site research-related testing were obtained on enrollment. At a single time point during convalescence (2–4 weeks after enrollment), patients provided a second serum sample. Home visits were attempted if patinets did not return but could be located.

### Samples

Sera were stored promptly on site at -80°C and shipped on dry ice. Paired sera were tested by ELISA and PCR to confirm and serotype acute dengue infections at UNC, USA.

### Serology for dengue

**IgM ELISA**. Dengue IgM capture ELISA was performed as previously described [14] with minor technical changes [15]. **IgG ELISA**. Dengue IgG ELISA was done as published earlier [16]

### Serologic interpretation

We used PRNT-validated ELISA cutoffs as we have previously [15]. We defined **acute dengue** as either IgG seroconversion (acute optical density [OD] <0.20 and convalescent OD ≥0.20) or a significant increase in antibody titer (convalescent IgG OD ≥0.30 or IgM OD ≥0.20 than acute). **Acute primary (first episode)** and **acute secondary (recurrent) dengue** were distinguished by the absence or presence of IgG (OD <0.35 and ≥0.35, respectively) in acute sera in those with acute dengue. Presence of IgG without a significant increase in titer defined **past dengue**. Cross-sectional **seroprevalence** at enrollment was the presence of IgG (OD ≥0.20) in acute sera; others were **seronegative**. For each ELISA assay we used two negative control human sera and each control sample was tested in duplicate. Positive cut-offs were determined during assay validation, and were based on the mean OD for negative control sera plus 2 standard deviations.

### PCR for dengue

We used 25 to140 μL of acute sera to confirm and serotype dengue as previously described [17], [15].

### Statistical analysis

We compared proportions by the χ^2^ test or Fisher’s exact test and continuous variables by Student t-test or the Wilcoxon rank sum test if not normally distributed. We also performed bivariable and multivariable logistic regression. Analyses were completed with Stata IC 11.0 (StataCorp LP, College Station, TX, USA).

## Results

### Patient Characteristics

Of 825 consecutively enrolled patients, 740 (90%) with paired sera were tested for dengue. The likelihood of a subject returning for convalescent serum sampling and clinical follow-up did not differ by age (p = 0.40), sex (p = 0.89), or self-reported urban vs. rural residence (p = 0.59). The reported median distance from residence to hospital was 2 km (interquartile range [IQR] 2–20) for those who followed up versus 3 km (IQR 2–30) for those who did not (p = 0.08). Among the 740 patients, the median age was 10 years (IQR 3–29). The median age did not differ between those who reported urban (9 years [IQR 2, 31]) vs. rural residence 11 years [IQR 4, 30], p = 0.33. Slightly more were male (52.6%), and overall, males were younger than females (median age 9 vs. 12 years, p = 0.007). The median reported duration of fever was 2 (IQR 1-4) days and of illness 3 days (IQR 1-5). Many (30.0%) reported taking an antibiotic before presentation. The median interval between acute and convalescent follow-up was 15 days (IQR 14–28).

### Laboratory Diagnosis of Acute Dengue

Of the 740 with dengue testing on paired sera, time to convalescent follow-up did not differ. Thirty-eight (5.1%) patients had acute dengue (29 recurrent [secondary] and 9 primary). More than half (441, 59.6%) had evidence of past dengue whereas 261 (35.2%) were seronegative for dengue. In sum, 470 (63.5%) were seropositive for dengue at enrollment (acute-phase serum IgG positive), since this group included those with acute secondary dengue and those with past dengue. Among 37 patients with acute dengue and PCR results (1 had insufficient sample for testing), 9 (24.3%) were PCR positive; 3 were DENV-1 and 6 were DENV-2. The median duration of illness was shorter for those PCR-positive (median 2 days [IQR 2, 3]) vs. PCR-negative (4 days [IQR 2, 12], p = 0.03). Adults had almost exclusively secondary dengue (94.1%), whereas 38.1% of acute dengue was primary dengue in children (p = 0.02).

### Epidemiologic Features of Acute Dengue

**Fig 1:**
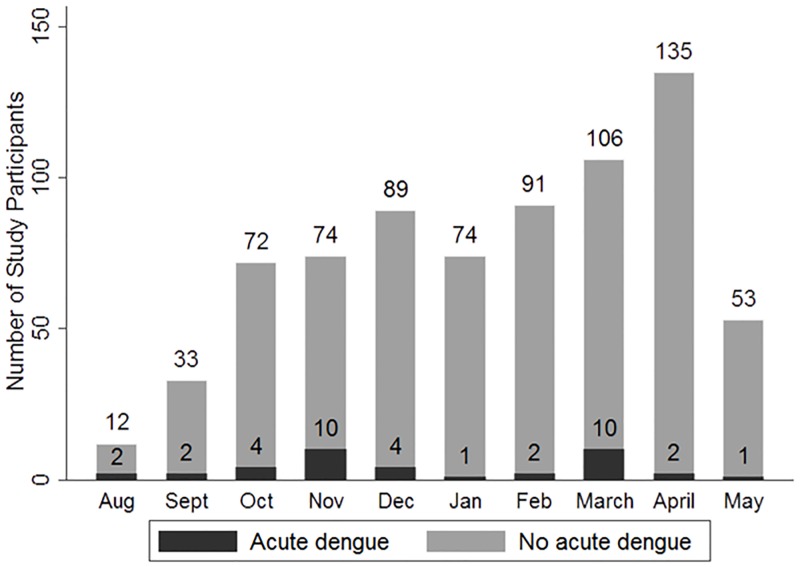
Temporal distribution of cases of acute dengue versus other febrile illness, Nicaragua.

**Fig 2:**
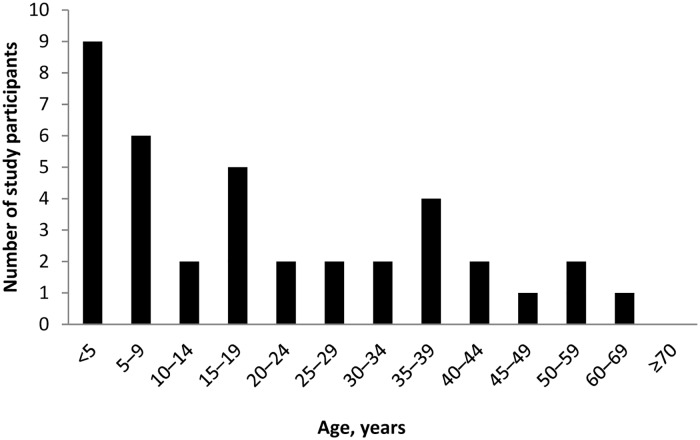
Age distribution of patients with acute dengue versus other febrile illness, Nicaragua.

The proportion of acute febrile illness (AFI) caused by dengue varied by month (Fig 1) from a high of 16.7% in August to a low of 1.4% of AFI in January (p = 0.002). Cases of acute dengue occurred throughout the year. The odds of acute dengue (1.04 [5% CI 0.48, 2.24], p = 0.92) was not higher in the rainy season (May to October) vs. the dry season (November to April). Acute dengue occurred most often in children and young adults of childbearing age (Fig 2). As shown in Table 1, however, patients with acute dengue did not differ from those with other febrile illness by age, sex, education, or occupation. Although a minority of patients (27.8%) reported rural residence, those with acute dengue did so more frequently and patients with acute dengue more often had farm animal (pig, cow, goat, horse) exposure (50.0 vs 29.5%, p = 0.007) and drank well or river water. Rural residence, farm animal exposure, and drinking well or river water were all highly associated. Those reporting both rural residence and farm animal exposure were at the highest risk of acute dengue (OR 2.9 [95% confidence interval (CI) 1.5, 5.9], p = 0.002). Risk was also increased with farm animal exposure (OR 2.4 (95% CI [1.2, 4.6])), drinking well or river water (OR 2.3 (95% CI [1.2, 4.6]), or rural residence (OR 2.1 (95% CI [1.1, 4.0]) as single factors.

**Table 1:** Epidemiologic characteristics of patients with acute dengue vs. other acute febrile illness, Nicaragua.

Demographic Characteristics	Acute dengue (n = 38)	No acute Dengue (n = 702)	OR (95% CI); *P* value unless noted otherwise
Median age, years, (IQR)	16 (6,35)	9 (3,28)	*P* = 0.26
Adult (≥ 18 years)	17 (45%)	267 (38%)	1.32 (0.68–2.54); 0.41
Male sex	18 (47%)	371 (53%)	1.24 (0.65–2.39); 0.52
Rural residence	16 (42%)	189 (27%)	2.06 (1.05–4.04); 0.03
Education, if age ≥18			*P* = 0.52
Illiterate	3 (18%)	37 (14%)	1.49 (0.76–2.91); 0.24^
Primary	8 (47%)	101 (38%)	
Secondary	6 (35%)	101 (38%)	
University	0 (0%)	27 (10%)	
Type of Work, if age ≥18^#^			*P* = 0.91
Home	11 (65%)	33 (50%)	1.05 (0.54–2.04); 0.89^
Student	1 (6%)	28 (10%)	
Worker	2 (12%)	51 (19%)	
Farmer	1 (6%)	17 (6%)	
Merchant	1 (6%)	18 (7%)	
Other	1 (6%)	20 (7%)	
Animal exposures			
Horse	14 (37%)	100 (14%)	3.51 (1.75–7.01);<0.001
Cow	10 (26%)	85 (12%)	2.59 (1.21–5.52);0.01
Pig	17 (45%)	156 (22%)	2.83 (1.46–5.49);0.002
Goat	5 (13%)	16 (2%)	6.49 (2.24–18.78); 0.001
Cat	11 (29%)	172 (25%)	1.25 (0.61–2.58); 0.54
Dog	30 (79%)	450 (64%)	2.09 (0.94–4.63); 0.07
Rodent	27 (71%)	493 (70%)	1.04 (0.51–2.13); 0.92
Swim/bathe/wade			
River	6 (16%)	78 (11%)	1.55 (0.63–2.82); 0.35
Other fresh water	1 (3%)	10 (1%)	1.91 (0.24–15.41); 0.54
Water source^#^			*P* = 0.03
Tap	22 (59%)	547 (78)	0.41 (0.21–0.81); 0.01^
Well	13 (35%)	144 (21%)	
River	1 (3%)	5 (1%)	
Bottle/boiled	1 (3%)	4 (1%)	

^#^ Does not sum to 100% secondary to rounding.

^ Reference group versus all other categories.

Notably, those with acute dengue who reported rural residence tended to be older than those who reported urban residence (median age 23.5 years [IQR 12, 35.5] vs. 10 years [IQR 2, 32], respectively, p = 0.06). Rural residence was more common in adults with acute dengue vs. other febrile illness (58.8% vs. 27.7%, p = 0.006) but not in children (30% vs. 26.5%, p = 0.73). Similarly, farm animal exposure and drinking well or river water were associated with acute dengue in adults (64.7% vs 28.8%, p = 0.002 and 47.1% vs. 19.2%, p = 0.006, respectively) but not in children.

### Seroprevalence of dengue IgG

**Fig 3:**
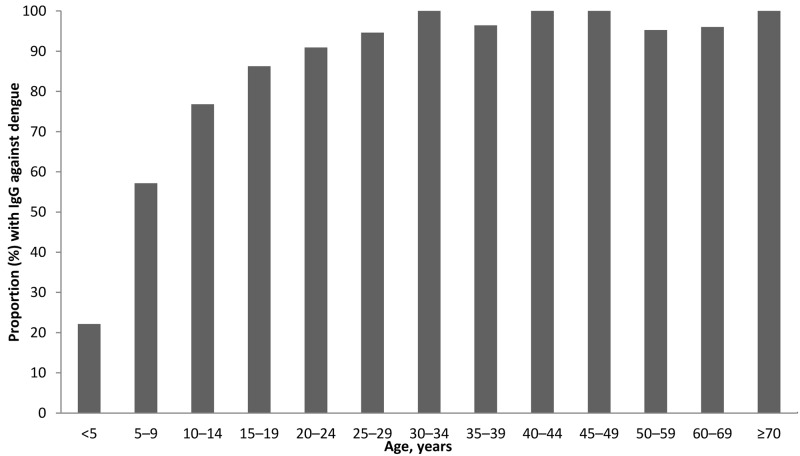
Proportion (%) of febrile patients with IgG against dengue, by age, Nicaragua.

Overall, 470 (63.5%) of patients were seropositive for dengue IgG at enrollment. The seroprevalance was 22% in those <5 years, 57% in those 5–9 years, 77% in those 10–14 years, 86% in those 15–19 years, 91% in those 20–24 years, 95% in those 25–29 years, and ~100% in those ≥ 30 years (Fig 3).

### Clinical Features of Acute Dengue vs. Other Febrile Illness (Table 2)

**Table 2:** Clinical characteristics of febrile patients with acute dengue vs. other acute febrile illness, Nicaragua.

Clinical Characteristics	Acute dengue (n = 38)	No dengue (n = 702)	OR (95% CI); *P* value* unless noted otherwise
Days ill, median (IQR)	4.0 (2.0, 7.0)	3.0 (1.0, 5.0)	*P* = 0.07
Days fever, median (IQR)	2.0 (1.0, 4.0)	2.0 (1.0, 4.0)	*P* = 0.66
Prior antibiotic treatment	10 (27%)	207 (30%)	0.86 (0.41–1.80); 0.68
Admitted to hospital	18 (53%)	260 (41%)	1.65 (0.83–3.30); 0.15
Symptom			
Headache	20 (53%)	352 (51%)	1.04 (0.55–1.96); 0.81
Chills	29 (78%)	437 (62%)	2.19 (0.99–4.86); 0.05
Sore throat	15 (39%)	201 (29%)	1.50 (0.78–2.85); 0.22
Cough	14 (37%)	275 (39%)	0.90 (0.46–1.77); 0.76
Dyspnea	3 (8%)	90 (13%)	0.58 (0.18–1.93); 0.38
Lethargy	5 (13%)	88 (13%)	1.06 (0.40–2.78); 0.91
Joint pain	18 (47%)	199 (29%)	1.98 (1.07–3.68); 0.03
Muscle pain	18 (47%)	218 (31%)	1.69 (0.93–3.08); 0.08
Abdominal pain	15 (39%)	179 (26%)	1.80 (0.94–3.47); 0.08
Vomiting	16 (42%)	252 (36%)	1.30 (0.67–2.51); 0.44
Diarrhea	8 (21%)	118 (17%)	1.32 (0.59–2.95); 0.50
Dysuria	6 (16%)	93 (13%)	1.13 (0.48–2.69); 0.78
Oliguria	7 (18%)	52 (7%)	2.82 (1.18–6.71); 0.02
Sign			
Conjunctival injection	0 (0%)	5 (1%)	*P* = 1.00
Pharyngeal exudate	12 (32%)	205 (29%)	1.12 (0.55–2.26); 0.76
Lymphadenopathy	3 (8%)	150 (22%)	0.31 (0.96–1.04); 0.06
Jaundice	1 (3%)	6 (1%)	3.13 (0.37–26.7); 0.30
Lung crackles	3 (8%)	79 (11%)	0.67 (0.20–2.25); 0.52
Tender spleen	0 (0%)	6 (1%)	*P* = 0.57
Tender liver	2 (5%)	18 (3%)	2.11 (0.47–9.44); 0.32
Hepatomegaly	1 (3%)	15 (2%)	1.24 (0.16–9.61); 0.84
Rash	5 (13%)	54 (8%)	1.52 (0.63–3.67); 0.35
Petechiae	3 (8%)	11 (2%)	5.38 (1.43–20.2); 0.01
Laboratory parameteter	Median (IQR)		
WBC per μl	9500 (6450–13100)	11800 (8600–16000)	*P* = 0.04
ANC per μl	6424 (4290–11250)	8562 (5754, 12038)	*P* = 0.08
ALC per μl	2099 (1139, 3180)	2350 (1508, 3654)	*P* = 0.19
Hemoglobin (g/dl) ^	11.2 (9.9, 12.3)	11.9 (10.8, 13)	*P* = 0.30
Platelets x 1000 per μl^^	230 (174, 271)	274 (216, 346)	*P* = 0.03

WBC, White blood count; ANC, Absolute neutrophil count; ALC, Absolute lymphocyte count; IQR, interquartile range; SD, standard deviation; Hb, Hemoglobin.

*Kruskal-Wallis test for proportions and skewed continuous variables; analysis of variance test: normally distributed continuous variables.

^217 with data.

^^ 400 with data.

Patients with and without acute dengue presented early (median 4 [IQR 2–7] vs. 3 days [IQR 1–5], p = 0.07). The duration of illness was shorter in those PCR-positive (median 2 [IQR 2–3] vs. median 4 [IQC 2–12] days, p = 0.03). Patients with acute dengue were as likely to have taken an antibiotic before presentation as others (27.0% vs. 30.2%, p = 0.68). Headache was similarly common (>50%) but those with acute dengue were more likely to report chills (78.4 vs 62.3%, p = 0.049), joint or muscle pain (47.4 and 28.6%, p = 0.01 and 47.4 vs. 31.4%, p = 0.04, respectively) and oliguria (18.4 vs. 7.4%, p = 0.015). Patients with acute dengue more frequently had a petechial rash (7.8% vs. 1.6%, p = 0.005) and lower white blood cell (median 9500 vs 11800, p = 0.04) and platelet counts (median 230,000 vs. 274,000, p = 0.03) but less frequently lymphadenopathy (7.9% vs. 21.5%, p = 0.04). The proportion hospitalized (52.9 vs. 40.5%, p = 0.15) and duration of hospitalization was similar (median 3.5 vs. 3 days, p = 0.85).

### Multivariable Modelling to Distinguish Acute Dengue vs. Other Febrile Illness

In a logistic regression model including an interaction term for adult and residence, there was not a statistically significant interaction between age (adult vs. child) and residence (rural vs. urban residence) (OR 3.13 [95% CI 0.77–12.73], p = 0.11). In a multivariable model in which potentially statistically significant (p≤0.10) epidemiologic and clinical features in bivariable analyses were evaluated for inclusion, rural residence with farm animal exposure (OR 2.9 [95% CI 1.4, 5.9], p = 0.003), joint pain (OR 2.2 [95% CI 1.1, 4.3], p = 0.02), and petechial rash (OR 5.4 [95% CI 1.4, 20], p = 0.01) were independently associated with acute dengue. An alternative final model included drinking river or well water, joint pain, and petechial rash (drinking river or well water OR 2.2 [95% CI 1.1, 4.5], p = 0.02, joint pain OR 2.2 [95% CI 1.1, 4.3], p = 0.02, and petechial rash OR 5.5 [95% CI 1.4, 20], p = 0.01). Rainy season and wading or swimming in fresh water were not associated with acute dengue when adjusted for rural residence with farm animal exposure or drinking river or well water. In a model including only clinical features, the presence of joint pain and petechial rash and the absence of lymphadenopathy independently predicted acute dengue (joint pain OR 2.2 [95% CI 1.1, 4.3], p = 0.02, petechial rash OR 5.2 [95% CI 1.4, 20], p = 0.02, and absence of lymphadenopathy OR 0.30 [95% CI 0.09, 0.997], p = 0.049). The clinical model was marginally less predictive (AIC 293) than the models including rural residence with farm animal exposure (AIC 283) or drinking river or well water (AIC 287).

### Clinical Diagnosis and Management of Acute Dengue

Physicians infrequently suspected dengue and infrequently inferred correctly its presence. Clinicians suspected dengue in only 3 of 34 (8.8%) patients with acute dengue for whom a clinical etiologic diagnosis was given and dengue was only confirmed in 3 of 16 (18.8%) patients thought to have dengue on enrollment (sensitivity 8.8% [95% CI 1.9–23.7] and specificity 98.0% [95% CI 96.6–98.9]) Of the 38 with confirmed acute dengue, most were thought to have focal bacterial infections (22); other recorded clinical diagnoses included febrile syndrome (6), leptospirosis (2), “viral not specified” (1), and bacteremia (1). Of note, 10 of 37 (27.0%) patients with acute dengue were treated with an antibiotic (missing data for 1), including one clinically suspected to have acute dengue.

## Discussion

We sought to identify and characterize dengue infection in a hospital-based cohort of children and adults with acute febrile illness in an understudied relatively rural region of Nicaragua. We prospectively enrolled consecutive patients with a reproducible criterion (documented fever) for nearly a year and emphasized convalescent follow-up to rigorously distinguish acute from past infections using paired serology in addition to confirmation by PCR. We comprehensively studied epidemiologic risk factors and clinical features to fully characterize acute dengue infections. We hypothesized that the median age of those with acute dengue would be shifted later (to adolescence) relative to what has been reported in urban Managua due to less intensive transmission. Additionally, we hypothesized that demographic and epidemiologic features associated with acute dengue might differ from those described previously. In our cohort, rigorous testing identified dengue as the etiologic agent responsible for 5.1% (38/740) of acute febrile illnesses. In addition to confirmatory testing by assaying paired sera for IgM and IgG, 24.3% were confirmed by PCR. A limitation of serology is that serologic cross-reactions occur between dengue and other flaviviruses. During this study, however, dengue was the only flavivirus known to be circulating in humans in Nicaragua. Japanese encephalitis and West Nile viruses are not known to occur, the area is not endemic for yellow fever nor is immunization offered, and Zika virus has only recently reached the Americas [13,18].

In our cohort presenting with acute febrile illness in León during 2008, we found dengue serotypes 1 and 2 caused illness throughout the year without distinct seasonality. In contrast, DENV-3 predominated in Managua during 2008–9, 2009–10, and 2010–11. [19]. In our study, acute dengue was associated with self-reported rural residence, contact with livestock, and drinking well or river versus tapwater. As expected, wading or bathing in river or other fresh water were not associated with acute dengue.

Dengue has traditionally been considered an urban and peri-urban disease, with the current pandemic attributed in part to widespread migration to cities [2]. However, recogition of the importance of dengue in semi-urban and rural areas is increasing [15,20]. Some studies have suggested that transmission at rural schools may play a role [21,22]. Additionally, lack of access to tap water and use instead of discarded water storage jars and concrete tanks have been implicated as important breeding sites for the vector [23], and mosquito populations may, therefore, be higher in some rural than urban areas [24]. A study in an agricultural settlement in Brazilian Amazonia linked local dengue to travel from high-risk urban areas [25]. In the Peruvian Amazon, *Aedes aegypti* geographic spread appears to be driven by human transportation networks along rivers and highways [26]. Since León is linked by highway to Managua, increased travel of persons or transport of mosquitos from Managua to rural communities surrounding Leon may in part explain our findings.

Notably, acute dengue was more common in adults reporting rural residence (per self-report of farm animal exposure, and drinking well and river water) vs. urban residence but not in children. We hypothesize that young adults in rural areas remain susceptible to symptomatic primary and secondary dengue infections (presenting as acute febrile illness) due to a lower intensity of infection relative to urban Managua. In contrast, most new dengue infections occur in children in urban areas, but a minority will present with acute febrile illness.

In León (population>200,000), we found a low (63.5%) overall seroprevalence of dengue compared with the 91% reported by Harris, et al. in the capital city of Managua (population > 1.3 million) [27]. The seroprevalance of dengue in our cohort increased with age and was 22% for ages <5 years, 57% for ages 5–9, 77% for ages 10–14, 86% for ages 15–19, 91% for ages 20–24, and ~100% for ages ≥ 30 years. In contrast, Harris and colleagues found that the seroprevalence in Managua was already 75% at age 4 and rose to 100% by age 16 [27].

In our cohort of predominantly school-aged children, adolescents, and young adults (overall median age 9 years [with 39% ≥18 years] and median age for acute dengue 16 years [IQR 6–35]) who sought care early in illness for undifferentiated fever, those with acute dengue had clinical features similar to those with other acute febrile illnesses. The only symptoms and signs independently associated with acute dengue vs. other acute febrile illness were presence of joint pain and a petechial rash, which was uncommon, and absence of lymphadenopathy. Despite the predominance of secondary rather than primary dengue (76.3 versus 23.7%, respectively), abdominal pain, hepatomegaly, jaundice, lethargy, and thrombocytopenia with hemoconcentration were rare, which is consistent with dengue without warning signs. Non-specific acute febrile illness caused by dengue in the Americas and elsewhere has been reported by others. In Puerto Rico in 2009, presence of a rash and hemorrhagic manifestations (e.g., petechiae) and absence of respiratory symptoms distinguished patients with acute dengue from those with acute influenza and other acute febrile illness [28].

In our study, only 8.1% of patients with dengue were identified clinically. Of the dengue cases correctly identified, 2 were children (1 primary and 1 secondary) and 1 an adult (secondary case). Similarly, we found the sensitivity of clinical diagnosis to be only 14% with relatively mild dengue characterized by absence of lymphadenopathy in rural and semi-urban southern Sri Lanka [15]. As in Nicaragua, the median age for those with acute dengue in the rural and semi-urban south of Sri Lanka was higher than that previously observed in the country’s largest city and capital Colombo [15]. In rural Cambodia, unsuscepted dengue was identified as a major cause of hospitalization and death in children, with delays in recognition and care-seeking contributing to its burden [23]. Our findings confirm the difficulties with clinical diagnosis, particularly in patients with recent onset of fever (median day 3) in the absence of a recognized dengue epidemic and in rural settings in which zoonotic infections may be suspected initially. Finally, clinical acumen is difficult to develop when confirmatory testing is generally not available, even in a subset of patients. This highlights the need for rapid, accurate point-of-care diagnosis [29], which could also limit frequent (27% of our patients with dengue) unnecessary antimicrobial use.

Our study does have some limitations. The proportion confirmed by PCR (24.3%) was lower than we observed previously (35.2%) [15] with similar enrollment criteria and testing methodology; however, as might be expected, the median duration of illness was shorter for those PCR-positive vs. PCR-negative. Additionally, since we did not do plaque reduction neutralization (PRNT) in this study, misclassification of other flaviviral infections as dengue is theoretically possible. West Nile and St. Louis encephalitis viruses may be transmitted in Nicaragua, since birds are the reservoir and the Pacific Americas flyway migration route goes through Nicaragua. However, we used an ELISA previously validated vs. plaque reduction neutralization (PRNT) and conclude that misclassification of dengue cases is unlikely. We had limited ability to evaluate rainfall and seasonality, since the study duration was just under a year rather than more than a year. Additionally, rural vs. urban residence was self-reported and we did not ask for similar classification of workplace; since *Aedes* mosquitos feed during the daytime, patients could have been exposed in other locations separate from where they live. Unique strengths of our study include rigorous confirmation of acute infection by World Health Organization criteria [30], a large sample size with 90% follow-up (critical to gold-standard diagnosis and assessment of outcomes) and prospective clinical correlation. We prospectively studied consequtively enrolled patients with an objective criterion (fever 38°C) using rigorous diagnostic criteria to minimize recall, selection, and diagnostic verification bias. We have not delineated the full clinical spectrum of dengue, which would require a prospective population-based study; however, we do describe symptomatic acute dengue prompting hospital-based care across a wide age span (1 year and older). We do believe that the population studied is representative of patients with symptomatic dengue in the region, since HEODRA has a large catchment area and is the only public teaching hospital. Patients with fulminant dengue may die before hospital evaluation, but most, including indigent patients from outlying areas, seek care there because of free access. Moreover, patients with dengue in this study presented early (median 2 days of fever and 4 days of illness).

In summary, we found that dengue is an important cause of undifferentiated fever in the less densely populated area of Western Nicaragua that surrounds León and that it strikes predominantly adolescents and young adults. The mild clinical illness we observed with dengue in this study mimics that reported with Zika virus infection [31]. Given that Zika virus is transmitted by the same vector (*Aedes aegypti*) and has been associated with severe congenital infection including microcephaly [32], the population we studied is clearly at high risk for being devastated by the Zika epidemic that has now reached Central America. Carefully conducted studies will be required to identify and distinguish these infections [13]. Epidemiologic and clinical studies of both dengue and Zika virus will now be complicated by their co-circulation in Nicaragua as well as elsewhere in Latin America. Studying patients early in illness and the determined pursuit of convalescent sera will maximize the likelihood of correctly diagnosing cases of dengue, Zika, and potentially dengue-Zika co-infections by PCR and paired serology. Owing to serological cross-reactivity between flavivruses, PCR testing of samples obtained early in illness is crucial. Since many low-income countries continue to lack the infrastructure and resources to conduct state-of-the-art laboratory testing in real-time, new point-of-care tests, prospectively studied and validated in cohorts such as that described here, are urgently needed.

## Supporting Information

S1 Checklist STROBE Checklist. (DOC)
